# Attempted Suicide with Adrenalin

**Published:** 1918-05

**Authors:** 


**Attempted Suicide With Adrenalin.** Raymond Grasset, Le Prog. Med., Oct. 20, 1917, reports the case of a druggist's chore-boy with advanced pulmonary tuberculosis. He took 15 grams of a 1:1000 solution of adrenalin and, 5 minutes later a second dose of 20 grams. Somewhat later, he took a third poison, probably scammonium, in unknown dose. Two hours later, he admitted the attempt at suicide and was given an emetic, bled and given inhalations of amyl nitrite. The principal symptom was marked stiffness of the neck. Later he received injections of caffeine and camphorated oil and lavage with chloral on account of persistent agitation. He recovered completely in two days.

